# Uga3 influences nitrogen metabolism in *Saccharomyces cerevisiae* by modulating arginine biosynthesis

**DOI:** 10.15698/mic2025.06.851

**Published:** 2025-06-12

**Authors:** Nicolás Urtasun, Sebastián Aníbal Muñoz, Martín Arán, Mariana Bermúdez-Moretti

**Affiliations:** 1 Universidad de Buenos Aires. Facultad de Ciencias Exactas y Naturales. Departamento Química Biológica. Buenos Aires, Argentina - CONICET. Instituto de Química Biológica de la Facultad de Ciencias Exactas y Naturales (IQUIBICEN). Buenos Aires, Argentina.; 2 Departamento de Ciencias Básicas y Experimentales, Universidad Nacional del Noroeste de La Provincia de Buenos Aires (UNNOBA), Junín, Buenos Aires, Argentina.; 3 Fundación Instituto Leloir e Instituto de Investigaciones Bioquímicas de Buenos Aires (IIBBA) - CONICET, Patricias Argentinas 435 (C1405BWE), Buenos Aires, Argentina.; a These authors contributed equally to this work.

**Keywords:** nitrogen metabolism, proline catabolism, arginine biosynthesis, Saccharomyces cerevisiae, Uga3 transcription factor

## Abstract

Nitrogen metabolism in *Saccharomyces cerevisiae* is tightly regulated to optimize the utilization of available nitrogen sources. Uga3 is a known transcription factor involved in the gamma-aminobutyric acid (GABA) pathway; however, its broader role in nitrogen metabolism remains unclear. Here, we demonstrate that Uga3 influences arginine biosynthesis, linking its function beyond GABA utilization when cells grow with proline as the sole and poor nitrogen source. Using a combination of intracellular amino acid quantification, proteomics, and gene expression analysis, we show that the absence of Uga3 leads to a significant increase in intracellular arginine levels and the up-regulation of *ARG5,6*, a key gene in the arginine biosynthesis pathway. Proteomic analysis of *uga3*∆ cells reveals differential expression of multiple nitrogen metabolism-related proteins, suggesting a broader regulatory role for Uga3. Surprisingly, chromatin immunoprecipitation (ChIP) assays indicate that Uga3 does not directly bind the *ARG5,6* promoter, implying an indirect regulatory mechanism. These findings expand the known functions of Uga3, positioning it as a key player in the coordinated regulation of nitrogen metabolism. Given the impact of nitrogen availability on industrial fermentation processes, our results provide new insights into optimizing yeast performance under nitrogen-limited conditions.

## Abbreviations

DCW - dry cell weight*,*

GABA - gamma-aminobutyric acid,

NCR - nitrogen catabolite repression.

## INTRODUCTION

*Saccharomyces cerevisiae* is a versatile model system for eukaryotic organisms, capable of utilizing a wide variety of nitrogenous compounds found in nature. This yeast can detect both the availability and type of nitrogen sources in the environment and rapidly modulate its transcriptional, metabolic, and biosynthetic machinery for their utilization 1,2,3,4,5. The internalization of amino acids and other nitrogenous compounds is facilitated by multiple permeases. Highly regulated and conserved pathways govern the sensing and metabolism of these sources 6. These compounds can either serve as building blocks for biosynthetic pathways or be degraded to ammonium and glutamate, two central intermediates in nitrogen metabolism 5.

Nitrogen availability is a critical factor for the biotechnological applications of this model yeast. During industrial fermentation processes, particularly in wine and beer production, nitrogen availability regulates yeast biomass formation and, consequently, the overall duration of the process 7. Furthermore, the production of numerous volatile compounds that contribute to flavor, including alcohols, short- to medium-chain fatty acids, and their ethyl or acetate ester derivatives depends on the presence of specific amino acids that serve as direct metabolic precursors for their synthesis 7,8,9,10. The internalization, biosynthesis, and accumulation of certain amino acids also play a significant role in stress tolerance during industrial fermentation. Multiple studies have documented the role of various amino acids, especially arginine and proline, in adaptation to temperature and pH fluctuations, oxidative stress, osmotic stress, and ethanol inhibition during fermentation processes 11,12,13,14.

Accordingly, understanding the hierarchical utilization of nitrogen compounds is essential for elucidating global regulatory processes and optimizing the industrial fermentation performance of *S. cerevisiae*. The type and quantity of nitrogen-related metabolites -particularly at the end of fermentation, when non-preferred nitrogen sources remain- can influence the organoleptic properties of the final product 8,9,15.

In the presence of preferred nitrogen sources, genes involved in utilizing non-preferred sources are not expressed due to nitrogen catabolite repression (NCR). This extensively studied mechanism allows yeast to utilize preferred nitrogen sources first, while repressing the use of non-preferred ones until the preferred sources are depleted. Once preferred sources are exhausted, NCR-regulated genes are de-repressed, enabling cells to utilize poorer nitrogen sources 5,16. While global and specific transcription factors have been identified as binding to the promoters of these genes, the interactions between global and pathway-specific regulators remain poorly understood. Furthermore, recent evidence suggests the existence of a hierarchy in the utilization of non-preferred nitrogen sources 17.

Dal81 is a transcription factor involved in the regulation of multiple pathways for the utilization of poor nitrogen sources, including gamma-aminobutyric acid (GABA; *UGA* genes) 18,19, urea and allantoin (*DUR* and *DAL* genes) 20,21, and various amino acids (*AGP1* gene) 22. Studies indicate that Dal81 interacts with other specific transcription factors to achieve pathway-specific regulation. For instance, Dal81 requires Uga3 for its regulatory role in the GABA pathway 23,24. Uga3 functions as a bridge between Dal81 and the promoters of the *UGA* genes. The induction of GABA catabolism genes decreases when other extracellular amino acids, either individually or in mixtures, are added to the medium 23.

Uga3 is a zinc cluster transcription factor originally characterized as a specific activator of genes involved in the GABA catabolic pathway, namely *UGA4*, *UGA1*, and *UGA2* 19,25. These genes contain CGG triplet motifs in their upstream activating sequences, which are recognized by Uga3 via its zinc finger domain spanning residues 17 and 44 19,23,25,26,27. A nuclear localization signal has been identified between residues 55 and 62, and its C-terminal acidic domain shares features with activation domains of other zinc cluster proteins 25. Uga3 interacts functionally with the general transcription factor Dal81, which is necessary for the hierarchical and coordinated expression of several permease-encoding genes, including those for GABA, leucine, and allophanate transport 22,28,29. In addition to its established role in GABA metabolism, recent evidence has shown that Uga3 positively regulates *BAP2*, a gene encoding a branched-chain amino acid permease, in response to extracellular leucine, but not its paralog *BAP3* 17. This regulation occurs through direct interaction with the *BAP2* promoter, suggesting that Uga3 may have broader functions in nitrogen regulation beyond the GABA pathway. These findings led us to hypothesize that Uga3 could participate in the transcriptional hierarchy that governs the utilization of poor nitrogen sources in *S. cerevisiae*.

In this study, proteomic and gene expression analysis of a *uga3*Δ yeast strain indicate that the *ARG5,6* gene, essential for arginine biosynthesis, is modulated by Uga3, using proline as a sole and non-preferred nitrogen source. This finding suggests that Uga3’s influence extends beyond the GABA pathway, playing a broader role in nitrogen metabolism. Moreover, our results indicate that Uga3 functions independently of Dal81, its established partner in *UGA* gene induction, further supporting its contribution to the global regulation of nitrogen utilization. Elucidating this novel role of Uga3 provides valuable insights into the hierarchical and coordinated control of nitrogen metabolism in *S. cerevisiae*, with substantial implications for both fundamental biology and biotechnological applications, particularly in the final stages of industrial fermentation, when non-preferred nitrogen sources remain.

## RESULTS AND DISCUSSION

**Figure 1:**
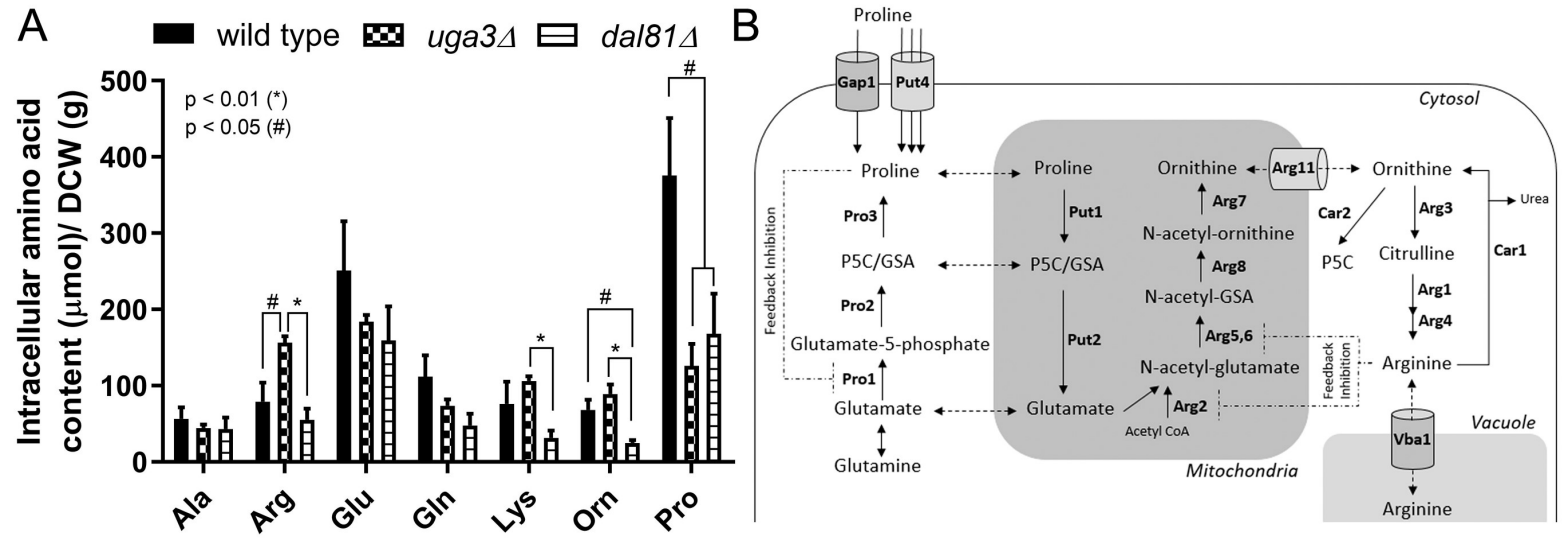
FIGURE 1: Intracellular amino acid content and metabolic pathways involved. **(A)** Intracellular content (µmol) per gram of dry cell weight (DCW) of alanine (Ala), arginine (Arg), glutamate (Glu), glutamine (Gln), lysine (Lys), ornithine (Orn), and proline (Pro) were determined in wild-type, *uga3*Δ and *dal81*Δ yeast strains. Amino acids were measured in cells harvested from cultures grown to an optical density at 600 nm (OD_₆₀₀_) of 1. Statistically significant differences were analyzed by one-way ANOVA followed by Tukey´s test. **(B)** Metabolic pathways of proline and arginine in *S. cerevisiae*. Protein names: Gap1, general amino acid permease; Put4, proline-specific transporter; Pro1, γ-glutamyl kinase; Pro2, γ-glutamyl phosphate reductase; GSA: glutamate-γ-semialdehyde; Pro3, Δ^1^-pyrroline-5-carboxylate (P5C) reductase; Put1, proline oxidase; Put2, P5C dehydrogenase; Arg2, N-acetyl-glutamate synthase; Arg5,6, N-acetyl-glutamate kinase and N-acetyl-glutamyl-5-phosphate reductase; Arg8, N-acetyl-ornithine aminotransferase; Arg7, N-acetyl-ornithine acetyltransferase; Arg11, ornithine transporter; Arg3, ornithine carbamoyltransferase; Arg1, argininosuccinate synthetase; Arg4, argininosuccinate lyase; Car1, arginase; Car2, ornithine aminotransferase; Vba1, vacuolas basic amino acid transporter. Activity of Pro1 or Arg2 and Arg5,6, is subject to feedback inhibition by Pro or Arg, respectively.

To gain deeper insights into the molecular mechanisms governing the coordinated utilization of poor nitrogen sources, we analyzed the intracellular content of 19 amino acids in wild-type cells and mutants lacking the transcription factors Uga3 or Dal81, using proline as a sole and non-preferred nitrogen source. **Figure 1A** shows the results for the intracellular content (µmol) per gram of dry cell weight (DCW) of alanine, arginine, glutamate, glutamine, lysine, ornithine and proline. The intracellular content of proline was significantly decreased in *uga3*Δ and *dal81*Δ strains compared to wild-type cells. Proline was the most abundant intracellular amino acid in the wild-type strain (375.6 ± 75.68 µmol/gram of DCW), followed by glutamate (251.03 ± 64.54 µmol/gram of DCW) and glutamine (112.00 ± 27.61 µmol/gram of DCW). These results contrast with previous studies using ammonium as the sole nitrogen source, where glutamine and glutamate were the most abundant amino acids in yeast cells 30. Our experiments using proline showed increased internalization and catabolism of this amino acid**.** Interestingly, arginine biosynthesis was significantly up-regulated in *uga3*Δ mutant compared to wild-type and *dal81*Δ strains, with an intracellular content of 156.64 ± 7.96 µmol/gram of DCW. Additionally, ornithine levels were slightly increased in the *uga3*Δ mutant and decreased in *dal81*Δ mutant. Amino acids with intracellular content below 20 µmol/gram of DCW were not shown or analyzed in this study.

Notably, the accumulation of arginine and ornithine differs significantly between *dal81*Δ and *uga3*Δ strains, with increased levels in *uga3*Δ and decreased levels in *dal81*Δ. This finding highlights that Uga3 and Dal81 function independently and may even exert opposing regulatory effects on specific metabolic pathways.

**Figure 2:**
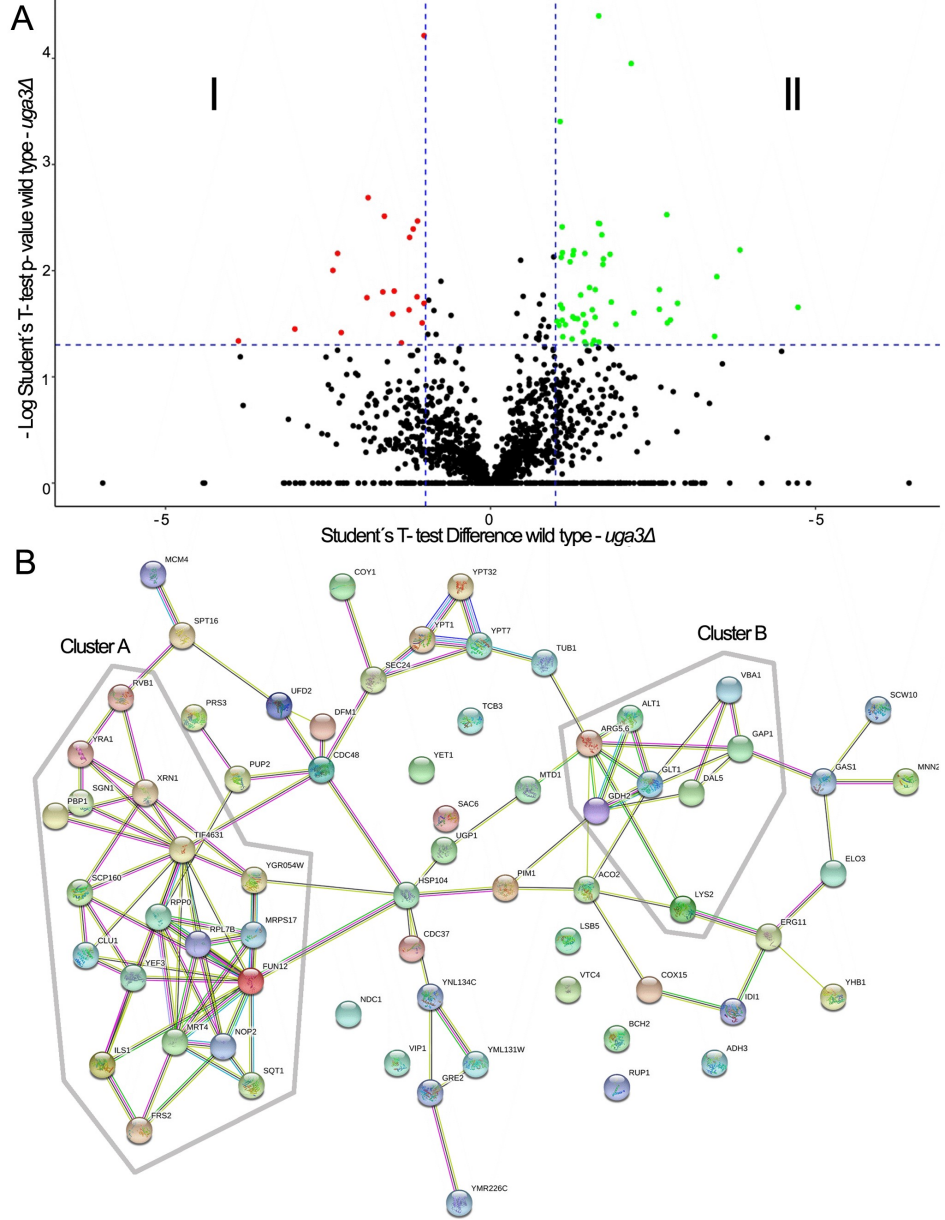
FIGURE 2: Comparative proteome analysis of wild-type and *uga3*Δ strains. **(A)** Relative protein expression in the *uga3*Δ strain compared to the wild-type. Volcano plot showing the statistical comparison of normalized protein abundances between wild-type and *uga3*Δ strains. Points in quadrants I and II represent proteins detected in both strains but exhibiting at least a 2-fold change (p < 0.05). **(B)** Network of interactions among differentially expressed proteins (both over- and under-represented) in wild-type and *uga3*Δ cells. Protein interaction networks were generated using the STRING web platform 32.

To further investigate the molecular mechanisms involved in the coordination of proline catabolism and arginine biosynthesis (**Figure 1B**), and based on the hypothesis that Uga3 participates in additional regulatory processes beyond the GABA-induced expression of the *UGA* genes, we compared the proteome of wild-type and *uga3*Δ cells. Proteomic analysis identified 1,750 proteins with high-confidence peptides and reliable fragmentation patterns. Comparison of normalized areas revealed differential expression of 67 proteins (p < 0.05, **Figure 2A**). Among them, 17 were over-represented in the *uga3*Δ mutant (**Figure 2A**, quadrant I), while 50 were under-represented (quadrant II). The identities and functions of these proteins are summarized in Tables S1 and S2. The absence of Uga3 led to altered expression of proteins with a significant diversity of functions. Using the PANTHER classification system software 31, we found that 20 of the 67 differentially expressed proteins are involved in nitrogen compound metabolic processes, with eight directly linked to amino acid metabolism. A STRING functional association analysis 32 revealed interactions among 61 of these proteins (**Figure 2B**) indicating biological relationships. Two main clusters emerged within the network: cluster A, consisting of RNA-binding proteins and ribonucleoproteins, and cluster B, which included enzymes involved in amino acid metabolism (Arg5,6, Lys2, Glt1, Gdh2, and Alt1) and amino acid transporters (Vba1, Gap1, and Dal5). Notably, several of these proteins (Gap1, Vba1, Gdh2, Glt1, and Dal5) are targets of NCR and are up-regulated under poor nitrogen conditions 33,34,35,36. *GAP1* encodes a general amino acid permease tightly regulated by both NCR and SPS-sensing pathways 37,38, while *VBA1* and *DAL5* function in vacuolar or secondary nitrogen source transport. *GDH2* and *GLT1* participate in glutamate metabolism and the GS-GOGAT cycle, respectively, linking nitrogen recycling and assimilation. Their co-occurrence suggests coordinated regulation under nitrogen limitation, in line with Uga3’s known role in NCR-sensitive gene expression. Although *ARG5,6*, *LYS2*, and *ALT1* are not classic NCR targets, they are responsive to amino acid availability and may be regulated by Gcn4 or other starvation-induced factors 3,39.

In parallel, we performed a similar analysis for Dal81 deficient cells. This pleiotropic transcription factor is involved in the coordinated utilization of poor nitrogen sources 29. Proteomic analysis of wild-type and *dal81*Δ cells identified 1,239 proteins with high-confidence peptides. Interestingly, Dal81 deficiency led to significant changes in only 12 proteins (data not shown), four of which are related to amino acid metabolism. STRING analysis revealed no significant functional associations. Given the known role of Dal81 in regulating numerous genes, the limited proteomic changes observed are likely due to the experimental conditions, in which proline was the sole nitrogen source. Dal81 function is often triggered by the presence of additional poor nitrogen sources, which were absent in our setup. These proteomic findings correlated with the few differences observed in intracellular amino acid content between wild-type and *dal81*Δ cells, except for proline and ornithine (**Figure 1A**).

As shown in **Figure 1**, the absence of Uga3 led to a significant decrease in intracellular proline content compared to wild-type strain. Conversely, the intracellular content of arginine (twice the wild-type levels) and ornithine (an intermediate in the arginine biosynthesis) increased. The intracellular amino acid profile and proteome analysis (**Figure 2**) suggest alterations in metabolic pathways. The significant increase in intracellular arginine content aligns with the observed over-representation of Arg5,6 (a key enzyme in arginine biosynthesis, **Figure 1B**). While our study quantified total intracellular amino acids, we did not distinguish their subcellular compartments. Amino acid compartmentalization may also impact cellular physiology. For instance, the yeast vacuole plays a critical role in nutrient storage, with arginine accumulating in the vacuole under nitrogen-replete conditions and mobilized to the cytosol during nitrogen scarcity. The import and export vacuolar arginine systems are inversely regulated by nitrogen availability 32. In *uga3*Δ cells, the vacuolar amino acid permease Vba1, responsible for arginine transport into the vacuole, was under-represented. This suggests that both the quantity and compartmentalization of arginine are altered in this mutant, though further studies are needed to confirm this hypothesis.

**Figure 3:**
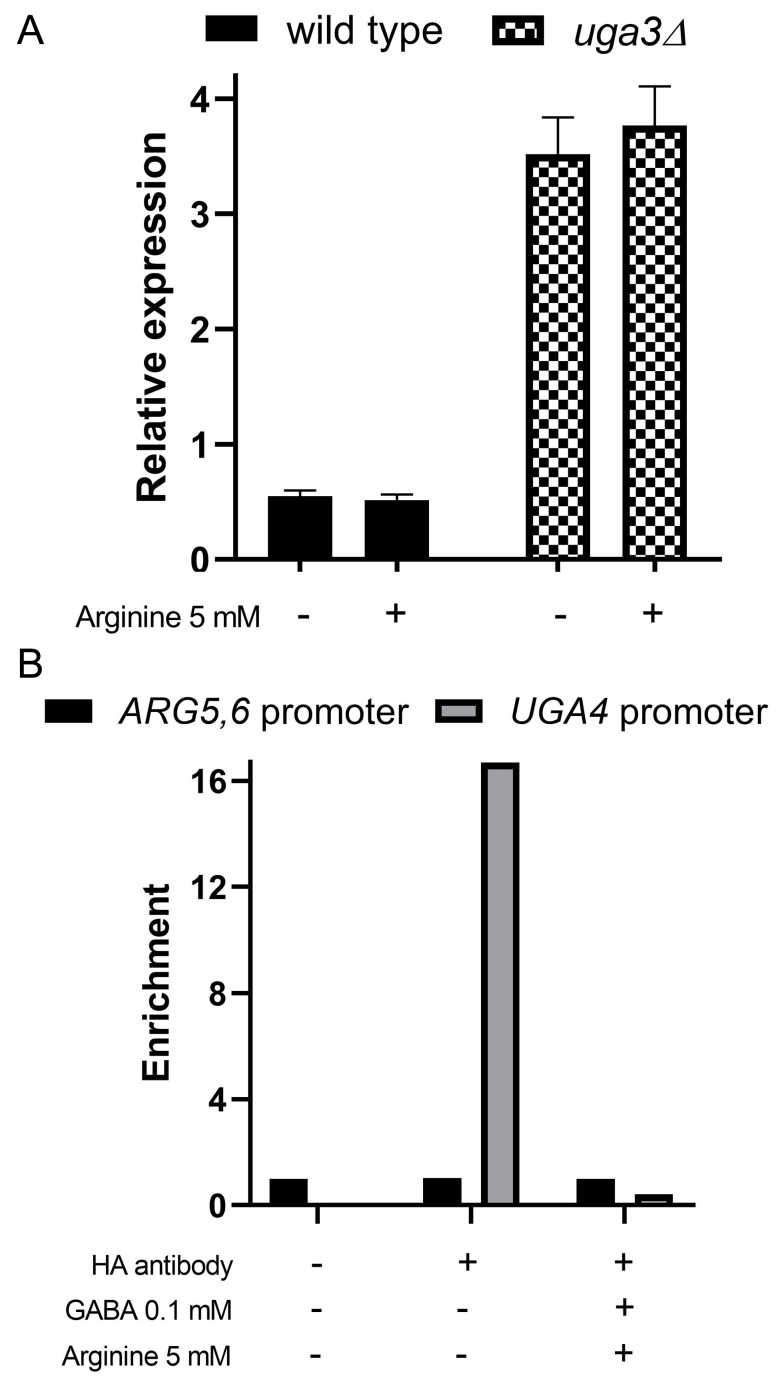
FIGURE 3: Gene expression analysis in wild-type and *uga3*Δ strains. **(A) **Effect of *UGA3 *deletion on arginine-mediated repression of the *ARG5,6 *gene. Wild-type and *uga3*Δ cultures were either not incubated or incubated with 5 mM arginine for one hour. Cells were then harvested, and total RNA was extracted. Reverse transcription was performed, followed by quantitative PCR (qPCR). *ARG5,6 *expression levels were normalized to the constitutively expressed *TBP1 *gene. **(B)**Analysis of the interaction of the Uga3 transcription factor with the *ARG5,6 *promoter. Cells expressing HA-tagged Uga3 protein (SBCY13 strain) grown in minimal medium (MM) were harvested and transferred to fresh medium with or without 5 mM arginine or 0.1 mM GABA followed by incubation for 1 hour. ChIP assays were performed using anti-HA antibodies. Quantitative PCR (qPCR) was conducted using primers targeting the *UGA4 *promoter region as a positive control and the *ARG5,6 *promoter region. Results are expressed as fold changes in Uga3 binding to the *UGA4 *or *ARG5,6 *promoter and represent the mean ± standard deviation of three independent experiments.

In *S. cerevisiae*, the *ARG5,6* locus encodes a transcript that is processed in mitochondria to produce two enzymes which form a complex with Arg2 and catalyze the second and third steps of arginine biosynthesis (**Figure 1B**). The transcription of *ARG5,6* and other genes of the pathway, is tightly repressed by arginine via the ArgR/Mcm1 transcriptional complex 16,40. Given this regulatory framework, further investigation is needed to understand how this mechanism operates under our experimental conditions. Since Uga3 is a transcription factor, we hypothesized that changes in Arg5,6 levels in *uga3*Δ cells could result from altered *ARG5,6* transcription. To test this, we assessed *ARG5,6* mRNA levels in wild-type and *uga3*Δ cells (**Figure 3A**). The expression of *ARG5,6* was significantly increased in the *uga3*Δ mutant, correlating with elevated Arg5,6 protein levels. Also, in correlation with under-representation in proteomic analysis, *VBA1* mRNA levels were decreased in the mutant (data not shown).

Interestingly, in our experimental conditions, *ARG5,6* expression in wild-type cells was not repressed by arginine, contrary to previous reports. This discrepancy likely arises from differences in nitrogen sources, as prior studies used ammonium, whereas we used proline. Nitrogen source quality profoundly affects the regulation of nitrogen metabolism genes. Furthermore, in the Σ1278b background, proline can serve as a precursor for arginine synthesis via up-regulation of *MPR1* and *PUT1* under specific stress conditions 41. This alternative pathway may contribute to the regulatory differences observed.

To determine whether the transcription factor Uga3 directly regulates *ARG5,6* expression by interacting with its promoter, we performed Chromatin Immunoprecipitation (ChIP) assays followed by qPCR. As shown in **Figure 3B**, no interaction between Uga3 and the *ARG5,6* promoter was detected under any tested condition. This suggests that the effect of Uga3 on *ARG5,6* regulation may be indirect, likely mediated by upstream changes in central nitrogen metabolism. Among known transcriptional regulators of *ARG5,6*, Gcn4 and the ArgR/Mcm1 complex represent the most likely candidates to mediate this effect 42*.* However, our previous proteomic analysis of a *gcn4*Δ strain under the same growth conditions revealed no significant change in *ARG5,6* expression compared to the wild-type strain 43, suggesting that Gcn4 is not involved in mediating the increased *ARG5,6* expression observed in the *uga3*Δ background. Furthermore, although the ArgR/Mcm1 complex is a known regulator of multiple arginine biosynthesis genes, only ARG5,6—among its typical targets—was differentially expressed in *uga3*Δ cells, while Arg1, Arg3, and Arg8 levels remained unchanged. These observations suggest that Uga3 influences *ARG5,6* expression through a regulatory mechanism distinct from currently known canonical pathways. Further studies are required to confirm this hypothesis and identify the potential transcriptional intermediates involved. Additionally, Arg5,6 is known to associate with nuclear and mitochondrial loci, influencing their transcription 44, and so, potentially amplifying the regulatory effects of Uga3 across other processes.

The integration of arginine biosynthesis and its regulatory networks with broader metabolic pathways underscores the interconnected nature of nitrogen metabolism in yeast. In cells deficient in Uga3, observed changes seem to extend beyond transcriptional regulation to mitochondrial function and vacuolar dynamics. These findings highlight the importance of studying compartmentalized nitrogen metabolism in different genetic contexts, providing insights into yeast adaptation to nitrogen-limited conditions.

Beyond nitrogen metabolism, arginine has been linked to stress resistance in various organisms 45,46,47,48. In baker's yeast, disruption of the *CAR1* gene, which encodes arginase —a key enzyme in arginine degradation— increases intracellular arginine levels and enhances freeze tolerance 46. Conversely, *CAR1* over-expression renders yeast strains highly sensitive to ethanol 12. Under oxidative stress, such as exposure to hydrogen peroxide or freeze-thaw cycles, yeast accumulates arginine, likely as part of a protective response 49,50. These findings emphasize the broader physiological significance of arginine metabolism beyond its canonical role as a proteinogenic amino acid. In addition, proline is a predominant amino acid in grape must and its accumulation negatively affects wine quality by contributing to the production of unpleasant flavors 10. In our study, the altered intracellular levels and compartmentalization of arginine observed in *uga3*Δ mutants, using proline as a nitrogen source, may influence stress responses and other cellular processes. Future studies should investigate whether these metabolic alterations in the *uga3*Δ background confer adaptive advantages or disadvantages under stress conditions. Elucidating this novel role of Uga3 provides valuable insights into the hierarchical and coordinated regulation of nitrogen utilization in *S. cerevisiae*, with important implications for both fundamental biology and biotechnological applications.

## MATERIAL AND METHODS

### Strains

The *S. cerevisiae *strains 23344c (*Matα ura3*), 26790 (*Mat*α *ura3 uga3*Δ) 51, SBCY17 (*Matα ura3 dal81*Δ*::natMX4*) and SBCY13 (*Matα ura3 6HA-UGA3*) 23, isogenic to the wild-type Σ1278b, were used in this work. Cells were grown in minimal medium containing 0.17% Difco yeast nitrogen base (YNB without amino acids and ammonium sulfate), 2% glucose as carbon source, 10 mM proline as nitrogen source and 0.002% uracil.

### Mass spectrometry analysis

Protein extraction and mass spectrometry analysis were carried out as already described 43,52. Briefly, total proteins were prepared by lysing yeast cells in 1.85 N NaOH, 7.5% of ß-mercaptoethanol on ice for 10 min, followed by precipitation using trichloroacetic acid (TCA) at a ﬁnal concentration of 8%. The TCA pellets were resuspended in sodium dodecyl sulfate (SDS) loading buffer. Extracts were subjected to SDS-PAGE. Protein digestion and Mass Spectrometry analysis were performed at the Proteomics Core Facility CEQUIBIEM, University of Buenos Aires/CONICET as follows: SDS-PAGE gel excised protein bands were reduced, alkylated in-gel digested with Trypsin. The recovered peptides were analyzed by HPLC-coupled mass spectrometry (Orbitrap QExactive technology coupled to nano-HPLC-ThermoScientific). The ionization of the samples was carried out using Electrospray. Mass spectra were analyzed with the Proteome Discoverer software (Thermo Fisher Scientific^™^), comparing against the *S. cerevisiae* strain ATCC 204508 S288c proteome. This comparison involved analyzing the total detected peptides and identifying the proteins present in the samples. For each protein identified based on high-confidence peptides with reliable fragmentation patterns, quantification was performed by calculating areas using Proteome Discoverer algorithms. Proteome Discoverer searches were performed with a precursor mass tolerance of 10 ppm and product ion tolerance to 0.05 Da. Protein hits were filtered for high confidence peptide matches with a maximum protein and peptide false discovery rate of 1% calculated by employing a reverse database strategy. In addition, the abundance of each protein was quantified based on the area calculation for each one. The areas for each identified protein were manually normalized and were statistically compared with Perseus software version: 1.5.8.5. 53. A protein was considered to be differentially expressed if it presented a relative change equal to or greater than two in its expression level, with a *p* value less than 0.05 with the Student's T-test.

### Determination of amino acids content

The intracellular concentrations of proline, glutamate, glutamine, arginine, lysine, ornithine, alanine, asparagine, glycine, serine, threonine, valine, isoleucine, histidine, leucine, tyrosine, phenylalanine, methionine and aspartic acid were determined as previously described 43. Briefly, cells were cultured in 25 mL of minimal medium at 28°C until the OD_600_ reached one unit. The cells were then centrifuged at 3500 x g for 10 min at 4°C. Cell pellets were washed twice with PBS buffer, resuspended in 2 mL ice-cold 80% methanol, disrupted by sonication and centrifuged at 4°C for 10 min at 15000 x g. Supernatants were collected and dried in a Savant SpeedVac (Thermo Scientific). Dried samples were solubilized in 0.5 mL sodium phosphate buffer (100 mM dissolved in D_2_O, pH = 7.4), supplemented with 2,2-dimethyl-2-silapentane-5-sulfonate-d_6_ (DSS, final concentration 0.33 mM) as chemical shift reference. All NMR experiments were performed at 298 K on a Bruker Avance III spectrometer operating at a proton frequency of 600.2 MHz. ^1^H-NMR 1D spectra were acquired using a standard Bruker 1D NOESY pulse program with pre-saturation during relaxation delay and mixing time, and spoil gradients (noesygppr1d). The NMR data were zero-filled, Fourier transformed, phase-corrected using NMRPipe and converted to a Matlab-compatible format for further processing and analysis. All spectra were referenced to DSS (^1^H δ = 0 ppm) and submitted to water peak elimination, baseline correction and normalization. The assignment was achieved using the freely available electronic databases HMDB and BMRB, and subsequently confirmed by 2D spectra including heteronuclear single quantum coherence (HSQC) and total correlation spectroscopy (TOCSY). 2D ^1^H-^1^H TOCSY spectra were collected with N1=512 and N2=2048 complex data points. The spectral widths for the indirect and the direct dimensions were 9615.4 and 9604.9 Hz, respectively. The number of scans per t1 increment was set to 36. The transmitter frequency offset was 4.7 ppm in both ^1^H dimensions. 2D ^13^C-^1^H HSQC spectra were collected with N1 = 512 and N2 = 2048 complex data points. The spectral widths for the indirect and direct dimensions were 24906.9 and 12019.2 Hz, respectively. The number of scans per t1 increment was set to 256. The transmitter frequency offset was 70 ppm in the ^13^C dimension and 4.7 ppm in the ^1^H dimension. The concentrations of the assigned metabolites were estimated using DSS as an internal reference standard. The total intracellular amino acid content (µmol) detected has been relativized to the amount of dry cell weight both estimated from the initial culture (25 mL, OD_600_=1). Statistically significant differences were analyzed by one-way ANOVA followed by Tukey´s test (*p < 0.01; # p < 0.05).

### Quantitative RT-PCR 

RT-qPCR experiments were performed according to Cardillo *et al*. 26. cDNAs were quantiﬁed by RT-PCR in a BIOER Linegene 9660 cycler. cDNAs were subsequently quantified by real time PCR using a BIOER Linegene 9660 cycler with specific primers (Fw-*ARG5,6*: CGCTTCCTGCTTGGCATTTT; Rv-*ARG5,6*: TACGGCCATTGTGTGCTCAT). Expression values correspond to the ratio of concentrations of specific mRNAs over *TBP1* (Fw-*TBP1*: TATAACCCCAAGCGTTTTGC; Rv-*TBP1*: GCCAGCTTTGAGTCATCCTC) determined in each sample. Results are expressed as the mean ± SEM of three independent experiments.

### Chromatin immunoprecipitation assays 

Chromatin immunoprecipitation (ChIP) experiments were performed according to Cardillo *et al. *28. Normal mouse IgG (Santa Cruz) or monoclonal anti HA antibody (HA probe (F-7), Santa Cruz) were used. Real time quantitative PCR was carried out in a BIOER Linegene 9660 cycler with primers amplifying the promoter regions of the *ARG5,6* gene (F-*ARG5,6-*qPCR CTGTGGCCGAATGGTGGTAA; R-*ARG5,6-*qPCR GGATAGCGAACAACAACACGC). A pair of primers amplifying a region of the *TBP1* gene (F-*TBP1-*qPCR TATAACCCCAAGCGTTTTGC; R-*TBP1-*qPCR GCCAGCTTTGAGTCATCCTC) was used as an unbound control. ChIP DNA was normalized to input DNA and calculated as a signal to noise ratio over an IgG control ChIP. The ΔΔCt method was used to calculate fold changes in binding to the promoter of interest 54. Results are expressed as the mean ± SEM of three independent experiments. Primers that amplify a portion of the *UGA4* regulatory region (F-*UGA4*-qPCR AATCGCTTATCGCTTATCGTG; R-*UGA4*-qPCR GGAACTGATTACTGTGCCAAG) and GABA-inducing conditions were used as a positive control for the ChIP.

## CONFLICT OF INTEREST

The authors declare no conflict of interest.

## SUPPLEMENTAL MATERIAL

Click here for supplemental data file.

All supplemental data for this article are available online at www.microbialcell.com/researcharticles/2025a-urtasun-microbial-cell/.
