# Supplementary material for: Uga3 influences nitrogen metabolism in Saccharomyces cerevisiae by modulating arginine biosynthesis
Source: Microb Cell. 2025 Jun 12;12:132–40. doi: 10.15698/mic2025.06.851 (PMC12186702; doi:10.15698/mic2025.06.851)
Supplement: Supplementary file 1 [file mic-12-132-s01.pdf]

Supplementary figures and tables

Table S1. Proteins under-represented in the *uga3Δ* compared to the wild-type strain.

	IDs	Nombre standard	Descripción
**1.**	P40319	ELO3	Fatty acid elongase involved in very long-chain fatty acid biosynthesis, sphingolipid biosynthesis and late endosome to vacuole transport via the multivesicular body sorting pathway; localizes to the endoplasmic reticulum.
**2.**	Q12213	RPL7B	Subunit of the cytosolic large ribosomal subunit; involved in maturation of the large subunit rRNA, biogenesis of the ribosomal large subunit, and translation.
**3.**	Q12680	GLT1	Glutamate synthase [NADH]; oxidoreductase activity, cellular amino acid metabolic process.
**4.**	Q03102		Uncharacterized membrane protein YML131W; UNKNOWN.
**5.**	P09436	ILS1	Cytosolic isoleucyl-tRNA synthetase that catalyzes isoleucyl-tRNA aminoacylation through the coupling of isoleucine to isoleucyl-tRNA.
**6.**	P06105	SCP160	mRNA binding protein involved in mRNA localization, chromatin silencing at silent mating type cassette and telomere, chromosome segregation and pheromone dependent signal transduction; localizes to cytoplasm, endoplasmic reticulum membrane and polysome.
**7.**	Q04301	VBA1	Vacuolar basic amino acid transporter.
**8.**	Q04951	SCW10	Endomembrane system, hydrolase activity, hydrolase activity, acting on glycosyl bonds, conjugation.
**9.**	P39676	YHB1	Flavohemoprotein; oxidoreductase activity, response to chemical, response to oxidative stress.
**10.**	Q12068	GRE2	NADPH-dependent methylglyoxal reductase; oxidoreductase activity, lipid metabolic process.
**11.**	P40086	COX15	Cytochrome c oxidase assembly protein; membrane, mitochondrial envelope, oxidoreductase activity, cofactor metabolic process
**12.**	P32558	SPT16	Subunit of the FACT, the primosome and the replication fork protection complexes; binds to histones and nucleosomes; involved in regulation of transcription, DNA replication and DNA replication independent nucleosome organization.
**13.**	Q03246	MRPS17	Component of the small subunit of the mitochondrial ribosome, which mediates translation in the mitochondrion.

**14.**	P33201	MRT4	Ribosome assembly factor. RNA binding, rRNA binding, RNA catabolic process, organelle assembly, rRNA processing, ribosomal large subunit biogenesis, ribosome assembly.
**15.**	P15625	FRS2	Ligase activity, cellular amino acid metabolic process, tRNA aminoacylation for protein translation.
**16.**	P30665	MCM4	Component of the MCM mini-chromosome maintenance complex.
**17.**	P52893	ALT1	Probable alanine aminotransferase, mitocondrial.
**18.**	P32500	NDC1	Structural component of the nuclear pore complex.
**19.**	P19145	GAP1	General amino-acid permease Transmembrane amino acid transporter of broad specificity, involved in uptake of extracellular amino acids; integral to plasma membrane.
**20.**	P31539	HSP104	Heat shock protein 104. Adenosine-binding protein chaperone involved protein folding, unfolding and refolding, cellular heat acclimation, and the inheritance of oxidatively modified proteins during replicative cell aging
**21.**	Q06685	VIP1	Bifunctional cytosolic inositol hexakisphosphate kinase involved in inositol phosphate biosynthesis and inositol pyrophosphate (PP-IP) dephosphorylation of IP7.
**22.**	P39730	FUN12	Eukaryotic translation initiation factor 5B Protein and ribosome binding GTPase involved in the formation of the cytoplasmic translation initiation complex and maturation of SSU-rRNA; involved in regulation of translational initiation; localizes to the cytosolic small ribosomal subunit and eukaryotic 48S pre initiation complex.
**23.**	P15365	DAL5	Allantoate permease. An allantoate transmembrane and dipeptide transporter; localizes to plasma membrane.
**24.**	P16521	YEF3	Translation elongation factor and ATPase involved in translational elongation and termination.
**25.**	P32861	UGP1	Uridylyltransferase activity involved in biosynthesis of beta-glucans, glycogen, and trehalose.
**26.**	P36775	PIM1	ATPase activity, hydrolase activity, peptidase activity, protein complex biogenesis, proteolysis involved in cellular protein catabolic process.
**27.**	P53912		Uncharacterized protein YNL134C oxidoreductase activity.
**28.**	Q12159	YRA1	RNA-binding protein and ATP-dependent RNA helicase inhibitor; involved in mRNA export from the nucleus and transcription-coupled nucleotide-excision repair.
**29.**	P35184	SQT1	Protein chaperone involved in biogenesis and assembly of the large ribosomal subunit.

**30.**	P07702	LYS2	L-aminoadipate-semialdehyde dehydrogenase involved in lysine biosynthesis.
**31.**	P53297	PBP1	mRNA binding protein; functions as a negative regulator of mRNA polyadenylation, and as a positive regulator of HO translation; involved in stress granule assembly and the maintenance of rDNA.
**32.**	P39935	TIF4631	Translation initiation factor involved in translational initiation, stress granule assembly, and ribosomal large subunit biogenesis.
**33.**	P53235		YGR054W. RNA binding, mRNA binding, translation factor activity, RNA binding, translational initiation.
**34.**	Q03940	RVB1	ATP dependent DNA helicase subunit of the nuclear Ino80 complex involved in chromatin remodeling, snoRNP assembly and regulation of transcription from RNA Pol II.
**35.**	P47075	VTC4	Calmodulin- and inositol hexakisphosphate-binding polyphosphate kinase involved in vacuolar transport, vacuole fusion, microautophagy and polyphosphate biosynthesis; localizes to endoplasmic reticulum and vacuolar membranes.
**36.**	P35723	YET1	Protein whose biological role is unknown; localizes to the endoplasmic reticulum.
**37.**	P36122	BCH2	Endomembrane system, molecular function, Golgi vesicle transport, carbohydrate metabolic process, cell wall organization or biogenesis.
**38.**	P40482	SEC24	Endomembrane system, membrane, Golgi vesicle transport, protein complex biogenesis, vesicle organization.
**39.**	P22147	XRN1	Chromatin-binding 5'->3' exoribonuclease that activates transcription initiation and elongation by RNA polymerase II; involved in nuclear mRNA catabolism and decay of non-functional rRNAs; role in processing of both rRNA and snoRNA; negative regulator of autophagy; localizes to the nucleus, cytoplasmic stress granules and mRNA P-bodies.
**40.**	P33327	GDH2	NAD-specific glutamate dehydrogenase Mitochondrial glutamate dehydrogenase involved in nitrogen metabolism.
**41.**	Q03640	TCB3	Cell cortex, cellular bud, endomembrane system, lipid binding, lipid metabolic process.
**42.**	P01123	YPT1	SNARE-binding GTPase involved in vesicle budding and docking, vesicle-mediated transport, endocytic recycling, macroautophagy, SNARE complex disassembly, and pre-mRNA catabolism; localizes to Golgi and ER membranes, pre-autophagosomal structures, and cytoplasmic vesicles.

**43.**	Q12743	DFM1	Endomembrane system, membrane, molecular function, proteolysis involved in cellular protein catabolic process, response to chemical, signalling.
**44.**	Q03690	CLU1	Cytoplasmic RNA-binding protein of unknown biological role; localizes to stress granules.
**45.**	P32379	PUP2	Subunit of 20S proteasome essential to pre-messenger RNA (mRNA) splicing; contributes to formation of catalytic spliceosome for second transesterification step.
**46.**	P40991	NOP2	Ribosomal RNA (rRNA) (cytosine-C5-)-methyltransferase; contributes to the maturation of LSU-rRNA from tricistronic rRNA transcript (SSU-rRNA, 5.8S rRNA, LSU-rRNA), rRNA base methylation, and 66S preribosome assembly.
**47.**	P54860	UFD2	Ubiquitin-ubiquitin ligase involved in the ubiquitin-dependent ERAD pathway for protein catabolism.
**48.**	P22146	GAS1	1,3-beta-glucanosyltransferase, catalyzes splitting and linking of (1-3)-beta-D-glucan molecules that leads to elongation of (1-3)-beta-D-glucan chains; involved in cell wall organization.
**49.**	P38069	MNN2	Endomembrane system, transferase activity, transferase activity, transferring glycosyl groups, carbohydrate metabolic process, protein glycosylation.
**50.**	P38689	PRS3	Subunit of a PRPP synthetase complex, which synthesizes 5-phosphoribose 1-diphosphate and is important for cell wall integrity.

Table S2. Proteins over-represented in the *uga3*Δ compared to the wild-type strain.

	**IDS**	**Nombre standard**	**Descripción**
**1.**	P51996	YPT32	Endomembrane system, membrane, mitochondrial envelope, GTPase activity, hydrolase activity, Golgi vesicle transport, endosomal transport, exocytosis.
**2.**	P25694	CDC48	ATPase, a component of several multiprotein ATPase complexes involved in the release of polyubiquitinated proteins; part of the ER-associated ubiquitin-dependent protein degradation system, stress-induced homeostatically regulated protein degradation (SHRED) pathway.
**3.**	P32599	SAC6	Actin filament binding protein involved in actin filament organization and formin-nucleated actin cable assembly.

**4.**	P34237	COY1	Protein whose molecular function is unknown; involved in vesicular transport within Golgi; localizes to the Golgi membrane.
**5.**	P32939	YPT7	GTPase involved in vacuole inheritance, regulation of non-autophagic vacuole fusion, retrograde (endosome to Golgi) transport, endocytosis, and the CVT pathway; also involved in macroautophagy and piecemeal microautophagy of the nucleus.
**6.**	P10614	ERG11	Lanosterol 14-alpha-demethylase; catalyzes the demethylation of lanosterol during the biosynthesis of ergosterol; localizes to the endoplasmic reticulum.
**7.**	P39533	ACO2	Putative mitochondrial aconitase isozyme; similarity to Aco1p, an aconitase required for the TCA cycle.
**8.**	P09733	TUB1	Tubulin complex subunit; involved in homologous chromosome segregation, mitotic sister chromatid segregation, and microtubule-mediated transport of the nucleus.
**9.**	Q01217	ARG5,6	Acetylglutamate kinase and N-acetyl-gamma-glutamyl-phosphate reductase involved in arginine, ornithine biosynthesis and regulation of transcription; localized to the mitochondrial matrix.
**10.**	P07246	ADH3	Mitochondrial NAD-dependent alcohol dehydrogenase involved in NADH oxidation.
**11.**	P06101	CDC37	Essential Hsp90p co-chaperone; necessary for passage through the START phase of the cell cycle; stabilizes protein kinase nascent chains and participates along with Hsp90p in their folding.
**12.**	P25369	LSB5	Protein involved in membrane-trafficking events at plasma membrane.
**13.**	P05317	RPP0	Large ribosomal subunit rRNA-binding constituent of the cytosolic large ribosomal subunit; involved in assembly of the large ribosomal subunit and translation.
**14.**	P15496	IDI1	Isomerase activity, lipid metabolic process.
**15.**	Q05016		YMR226C. NADP(+)-dependent serine dehydrogenase and carbonyl reductase; acts on serine, L-allo-threonine, and other 3-hydroxy acids.
**16.**	Q02046	MTD1	NAD-dependent 5,10-methylenetetrahydrafolate dehydrogenase; plays a catalytic role in oxidation of cytoplasmic one-carbon units
**17.**	P40561	SGN1	Cytoplasmic RNA-binding protein; contains an RNA recognition motif (RRM); may have a role in mRNA translation.
